# Preventing Type 2 Diabetes in Women With Gestational Diabetes: Three Theoretical Perspectives on Behavior Change

**DOI:** 10.1111/jmwh.13747

**Published:** 2025-03-20

**Authors:** Lotte Elton, Ann‐Kristin Porth, Julie L. O'Sullivan, Jan C. Zoellick, Paul Gellert, Andy Guise, Alexandra Kautzky‐Willer

**Affiliations:** ^1^ Department of Population Health Sciences, School of Life Course and Population Sciences Kings College London London United Kingdom; ^2^ Division of Endocrinology and Metabolism, Department of Internal Medicine III Medical University of Vienna Vienna Austria; ^3^ Institute of Medical Sociology and Rehabilitation Science, Charité – Universitätsmedizin Berlin corporate member of Freie Universität Berlin and Humboldt‐Universität zu Berlin Berlin Germany; ^4^ Einstein Center Population Diversity Berlin Germany

## INTRODUCTION

Gestational diabetes (GDM) affects 1 in 6 pregnancies worldwide^1^ and around a third of those diagnosed with GDM will develop Type 2 diabetes mellitus (T2DM) within 15 years.^2^ Follow‐up care for people with GDM therefore offers an opportunity to detect blood glucose abnormalities early and reduce the risk of T2DM complications.^2^ Recommendations for postpartum GDM care focus on glucose testing and health education for lifestyle changes.^3^ Guidelines recommend that people with GDM should undergo glucose testing with an oral glucose tolerance test or with hemoglobin A1c (HbA1c) at 6 to 12 weeks postpartum to exclude T2DM. HbA1c or fasting glucose testing at regular 1 to 3 year intervals is advised to identify progression to T2DM.^3^ People diagnosed with GDM should be offered lifestyle advice on weight control, diet, and exercise, which may be delivered via structured education programs.^3^

Although guidelines emphasize the importance of providing follow‐up care to people with prior GDM,^3^ this care is often lacking. A recent editorial in *The Lancet* highlights the slow progress made in implementing GDM follow‐up care, despite its potential to improve long‐term cardiometabolic health.^4^ Uptake of glucose screening is poor globally, with only a third of people with prior GDM receiving regular screening as recommended.^5^ Barriers to screening include a lack of clear guidance about T2DM risk, competing responsibilities such as work or childcare, and poor continuity between pregnancy and primary care services.^6^ Behavioral interventions for T2DM prevention have also had mixed success.^7^ Only 2 of 13 controlled trials regarding lifestyle interventions for people with prior GDM showed significant reduction in T2DM, and there is evidence of publication bias.^8^ All this suggests that postpartum glucose screening and education interventions have not had the desired effect.

Public health interventions are complex, involving multiple stakeholders and unpredictable outcomes. One way to understand and improve these interventions is by using theory: frameworks of ideas or concepts to explain how something works or why something happens. Theoretical frameworks can help policymakers and health care professionals understand the behavior of key stakeholders, identify appropriate strategies for change, and predict possible outcomes. By incorporating social, psychological, and behavioral factors, theory provides useful insight when developing new interventions or assessing why existing ones have not been successful. However, interventions for T2DM prevention in people with GDM often lack theoretical grounding, and few studies specify a theoretical framework for their intervention.^9^

This article examines challenges in GDM follow‐up and considers solutions by drawing upon 3 theoretical frameworks from health psychology and sociology: the Health Action Process Approach (HAPA),^10^ Link and Phelan's theory of stigma,^11^ and Bourdieu's social practice theory.^12^ Each theory offers a different but complementary perspective on the behavioral changes required for effective T2DM prevention. We offer suggestions for more robust, theoretically‐grounded models of GDM follow‐up care to improve postpartum metabolic health and reduce T2DM risk.

### HAPA

HAPA is a health psychology framework that explains how people transition from deciding to adopt healthier behaviors to maintaining them over time.^10^ The HAPA framework describes psychological constructs (ideas) that influence whether an individual is able to plan for, initiate, and maintain behavior change over 2 phases. In the motivational phase, people form the intention to change their behavior—for example, a person with GDM might decide to increase physical activity or cut out sugar‐sweetened drinks. Then, in the volitional phase, a person converts their intentions into action and must maintain these new behaviors over time.

In the motivational phase, the key constructs are a person's perception of the potential risks or benefits associated with a behavior and their confidence in their ability to successfully change it. To commit to postpartum screening, a person with GDM must perceive that not doing so might be risky and that some benefit will result. They must also feel confident they can make and attend a screening appointment. This confidence in one's own actions is known as *action self‐efficacy* and is especially important in the early stages of behavior change, when individuals need to build confidence to initiate change. Next, the volitional phase focuses on turning intentions into actions and sustaining them. This involves action planning (deciding when, where, and how to act) and coping planning (identifying and overcoming potential barriers). Sustaining behavior also requires confidence in maintaining changes over time (maintenance self‐efficacy) and resuming them after relapses (recovery self‐efficacy).

Elements of HAPA like action self‐efficacy and coping planning can help to predict long‐term behaviors in people with GDM.^13^, ^14^ Interventions to reduce T2DM risk should therefore address the psychological constructs outlined in HAPA. To support people in building the intention to make lifestyle changes, the risk of developing T2DM and the possible benefits of lifestyle changes must be clearly explained. However, many people with GDM report planning to make lifestyle changes to prevent T2DM postpartum but facing barriers like time constraints, lack of childcare, low income, or stressful jobs.^15^ This suggests that people particularly struggle in the volitional phase of HAPA.

Health care professionals can support action planning in the volitional phase by helping people create detailed and achievable diet and exercise goals. Health care professionals can help people identify possible challenges to changing behavior and develop strategies to address them: for example, by recommending at‐home exercises or child‐friendly activities like walking if childcare is an issue. Barriers to postpartum testing may be addressed through flexible scheduling options, such as after‐hours laboratory services. Finally, regular follow‐up contact (at appointments, via email, or through apps) can help build self‐efficacy and encourage sustained behavior change.

### Link and Phelan's Theory of Stigma

Link and Phelan's theory of stigma is a sociological framework that explains how stigma arises and operates in society. The authors define *stigma* as the co‐occurrence of labeling, stereotyping, separation, status loss, and discrimination, often in the context of unequal power dynamics.^11^ Individuals are labeled according to differences, when people diagnosed with GDM are categorized as having high‐risk pregnancies.^16^ These labels are linked to undesirable characteristics: for example, people with GDM may be stereotyped as having poor eating habits or lacking willpower.^17^ Diagnoses like GDM can create social division by separating individuals into diabetes‐specific clinics, identifying them as different from their pregnant peers.^18^ This can result in status loss, lowering someone's social standing or esteem within society. The sociocultural value placed on pregnancy and motherhood means that people are particularly vulnerable to status loss where their pregnancy is complicated by a condition like GDM.^17^

Status loss goes hand‐in‐hand with discrimination, wherein labeled individuals are treated unfairly either socially or institutionally. In the case of GDM, people describe feeling ashamed about weight gain or not meeting blood glucose targets, and worry that health care providers assume they are not following dietary guidance.^16^ Many studies outline the impact of GDM stigma, with people with GDM reporting feelings of guilt, shame, self‐blame, doubt, and anxiety.^16^, ^17^ The effect of stigma is often compounded where people with GDM are stigmatized on the basis of other characteristics, such as being from a minoritized ethnic background.^17^

Perceived discrimination from health care professionals may make people feel ashamed or as though they have failed in their efforts to manage GDM. This may prompt them to disengage from health care services after birth and avoid regular diabetes screening.^17^ Similarly, people who associate dietary change, exercise regimes, or glucose testing with the shame they experienced during pregnancy may wish to forget GDM and associated health behaviors after birth.^16^ It is therefore essential that health care professionals pay careful attention to their approach to the care of people with GDM. When possible, training on stigma and its impact on health outcomes should be provided to those caring for people with GDM. Care providers should stress that GDM is a common condition caused by a complex interplay of genetic and hormonal factors and not the patient's fault.

### Bourdieu's Social Practice Theory

Social practice theory, developed by sociologist Pierre Bourdieu, explores how social structures and relationships shape individual behavior.^12^ Different types of resources (capital) influence a person's social position, including their financial resources (economic capital), social connections (social capital), knowledge and education (cultural capital), and reputation (symbolic capital). These forms of capital determine a person's ability to succeed or gain influence in society. Bourdieu also introduces the concept of *habitus*, a term which, unlike its medical use to describe body shape, refers to deeply ingrained patterns of thought and behavior shaped by an individual's social environment and access to capital. Differences in habitus can be so pronounced that it is difficult for people to understand the language, concepts, or practices of another social group, including their health beliefs and behaviors. Cultural and social norms often deepen social inequalities by valuing certain ways of thinking and behaving over others.

Possessing capital such as family support, ability to afford healthy food, or a high education level helps individuals follow health care advice and engage in health‐promoting behaviors. People who share similar traits (habitus) with their health care professionals can have more fluent exchanges, facilitating better care.^19^ Conversely, people who have little capital may face greater barriers to accessing health care services, have more difficulty communicating with health care professionals, and find it harder to follow health care advice. They therefore risk being interpreted as nonadherent to health care guidance and may be discredited in health care interactions. Moreover, people with little capital may struggle to implement lifestyle changes because they have fewer resources to help make them.

Many T2DM prevention strategies for people with prior GDM assume that patients can easily access health information, follow public health advice, and make the recommended lifestyle changes. These strategies are therefore more likely to succeed in those with high social capital. Although health care professionals have little power on their own to change societal inequalities, they should ensure that health care guidelines do not privilege certain groups. When guidelines overlook the needs of marginalized patients, health inequities can worsen. It is therefore important to consider how the social and cultural contexts of patients might be incorporated into GDM follow‐up care. Those developing education interventions for T2DM prevention should be mindful of who might be excluded, such as those who do not speak the national language. Similarly, care pathways should be designed to prevent exclusion based on habitus. Dietary guidance, for example, can be adapted to be relevant to the local population, incorporating food types commonly eaten by different cultural groups.

## DISCUSSION

The 3 theoretical frameworks outlined here highlight different facets of behavior change. The HAPA model offers insight into how people may be supported to overcome individual barriers to behavior change but overlooks the social environment in which they make decisions. Social practice theory adds context by highlighting how an individual's beliefs, feelings, and behaviors are shaped by wider social structures. By using social practice theory, health care providers could understand the psychological factors outlined in HAPA (eg, *action self‐efficacy*) as dependent on access to resources. Stigma theory integrates individual and structural factors, demonstrating how the discrediting of certain individuals can shape their behaviors and affect their social position.

T2DM interventions relying on behavior change must help individuals to make these changes within the context of their lives, families, and social networks. GDM follow‐up care pathways should be accessible, designed to make people's participation in health care easy, and remove barriers to attending appointments or laboratory screening. Considerations might include on‐site childcare, automatic referrals from pregnancy care to primary care providers, and offering glucose testing in postnatal midwife or pediatric appointments. One good example is the American College of Obstetricians and Gynecologists’ recent recommendation that people with GDM should be offered the first postpartum glucose screening during the birth hospitalization (instead of 4‐12 weeks after) to maximize participation.^20^

Being persistently identified as at risk even after pregnancy may worsen the negative impact of GDM upon people's self‐image. Similarly, regular screening may exacerbate health anxiety and perceived stigma. The challenge is to develop effective interventions while ensuring that the recipients do not feel stigmatized. When possible, segregating people with GDM into separate antenatal or postpartum services should be avoided. GDM lifestyle education and glucose screening may be incorporated into postpartum primary care appointments, and T2DM prevention could take the form of affirmative health promotion for the whole family.^16^

It is crucial to consider patients’ habitus and access to resources when designing T2DM prevention programs. As a minimum, social determinants of health such as education level and socioeconomic status should be included in the evaluation of any T2DM prevention program to identify inequities in health outcomes or access to services. Many studies of lifestyle interventions fail to report or adjust for these factors.^21^ Those designing interventions or caring for people with GDM must consider stigma and social exclusion as key factors impeding the uptake of screening and the adoption of healthier behaviors.

The theories discussed here can support health professionals to understand patients’ behavior, including why they may not engage with T2DM prevention efforts, and to consider how patients can be better supported. T2DM prevention is a multidisciplinary responsibility that should be considered by primary care providers, midwives, and obstetricians alike, from the earliest stage of a pregnancy affected by GDM. Midwives are closely involved with people with GDM during pregnancy, yet are not mentioned in seminal reviews or guidelines on GDM care.^3^, ^15^ Accordingly, surveys have shown that only half of midwives screen patients with prior GDM during postpartum visits.^22^ Interventions for T2DM prevention should involve the entire perinatal interprofessional team and consider how health promotion can be embedded throughout pregnancy and postpartum care.

## CONCLUSION

When designing interventions to prevent T2DM in people with GDM, it is important to recognize that people's motivation and volition for behavior change are shaped by their experiences and sociocultural backgrounds. To be successful, interventions should acknowledge people's health beliefs and behaviors as part of longstanding social, cultural, and historical narratives. Health care professionals have an important role to play in T2DM prevention: for example, by helping individuals develop action plans specific to their sociocultural context or through advocating for access to culturally appropriate education resources.

Approaching public health interventions from a theoretical perspective allows policymakers and health care professionals to recognize the complex interplay of influences on health behavior and uncover the mechanisms most amenable to intervention. The 3 theoretical approaches we have presented offer insight into why GDM follow‐up care may be unsuccessful and provide suggestions for how postpartum care of people with GDM can be improved in future.

## CONFLICT OF INTEREST

The authors have no conflicts of interest to disclose.
